# The impact of early re-resection in patients with pT1 high-grade non-muscle invasive bladder cancer

**DOI:** 10.3332/ecancer.2012.269

**Published:** 2012-09-18

**Authors:** Nikhil Vasdev, Jose Dominguez-Escrig, Edgar Paez, Mark I Johnson, Garrett C Durkan, Andrew C Thorpe

**Affiliations:** Department of Urology, Freeman Hospital, Newcastle upon Tyne, UK

## Abstract

**Aim:**

To evaluate the impact of early re-resection on the incidence of tumour recurrence and progression in patients with pT1 high-grade non-muscle invasive bladder cancer (HG-NMIBC).

**Patients and methods:**

From 2001 to 2008, 486 consecutive patients were diagnosed with pT1 HG-NMIBC. Data were collected retrospectively which included patient demographics, histological parameters including the presence of detrusor muscle at initial TUR and at re-resection, adjuvant intravesical therapy, and recurrence and progression rates. Early re-resection was performed within six weeks of initial TUR. Patients comprised those who underwent an early re-resection (Group A, *n* = 172) and those who did not (Group B, *n* = 314).

**Results:**

At initial TUR, detrusor muscle was present in 61% (*n* = 105) of patients in Group A and 76% (*n* = 240) of patients in Group B. At early re-resection, detrusor muscle was present in 77.9% of cases. A residual tumour was present in 54.6% of re-resected cases. The overall incidence of tumour recurrence was 35% and 42% in Groups A and B, respectively. During follow-up, there was a significantly higher rate of tumour stage progression in patients who did not undergo early re-resection (Group B 14.4% vs. Group A 3.3%, *P* < 0.05).

**Conclusions:**

Early re-resection facilitates accurate staging and clearance of residual disease. Subsequent rates of tumour stage progression are significantly improved. We advocate early re-resection for all patients with HG-NMIBC.

## Introduction

Bladder carcinoma is the most common malignancy of the urinary tract. The worldwide age standardised incidence rate (ASR) is 10.1 per 100,000 for males and 2.5 per 100,000 for females. It is the 7th most common malignancy in men and 17th in women. Approximately 356,000 new bladder cancer cases (274,000 males and 83,000 females) occurred worldwide in 2002 [1]. In the United Kingdom (UK), 10,091 new cases (7,284 males and 2,807 females) diagnosed in 2007, representing 1 in every 29 new malignancies diagnosed each year in the UK (Cancer statistics, Cancer Research UK 2007).

When diagnosed urothelial carcinoma of the bladder presents as non-muscle invasive papillary tumours in 70–85% of cases [2]. Recurrence is common within this group ranging from 0 to 80%, and, more importantly, 10% of pTa tumours and 35% of pT1 tumours will progress to muscle invasive disease [3, 4]. Disease progression has been demonstrated to correlate with tumour size, multi-focality, tumour stage, grade, and early recurrence [2].

While transurethral resection of tumour (TUR) in combination with intra-vesical chemotherapy or immunotherapy remains the “gold-standard” in the management of non-muscle invasive bladder cancer (NMIBC), initial TUR may be insufficient at completely removing and staging the tumour, increasing the likelihood of tumour recurrence and progression. The incidence of residual tumour following initial TURBT in patients with high-grade non-muscle invasive bladder cancers (HG-NMIBC) can be as high as 33–53% [2, 3]. Additionally, 10% of initial resections are deemed to be under-staged [4, 5]. Current European Association of Urology (EAU) guidelines suggest that early re-resection may improve recurrence-free survival [1, 5, 6].

## Patients and methods

To assess our single institution (tertiary referral regional urology centre), experience of the clinical impact of early re-resection in patients with newly diagnosed HGNMIBC.

Between January 2001 and January 2008, 486 consecutive patients were diagnosed with pT1 high-grade/pTis urothelial carcinoma at initial TUR. Patients with pTa tumours and those with ≥pT2 tumours were excluded. Subset analysis was performed after dividing the cohort in two study groups: 172 patients who underwent an early re-resection, within six weeks from initial TUR (Group A) and 314 patients who did not undergo re-resection at the discretion of their urological surgeon (Group B).

The medical records and hospital electronic database were retrospectively reviewed and cross-referenced with the Pathology Department Tumour Registry. Data collected included patient demographics (age at diagnosis and gender), clinical and histological tumour characteristics at initial TUR, and re-resection as well as histological grade and stage of subsequent recurrences. The incidence of the presence or absence of detrusor muscle at initial TUR and at re-resection was recorded, respectively.

Tumours were staged according to the 1998 World Health Organization (WHO) and the International Society of Urological Pathology (ISUP) (1998 WHO/ISUP classification) and further staged published by the WHO in 2004 TNM classification. All specimens were analysed and cross-reported by at least two Consultant Uro-pathologists and reviewed at our Uro-Oncology Multi-Disciplinary Team meeting.

All patients underwent upper tract imaging consisting of an ultrasound and intravenous urogram (IVU) or a CT urogram at initial diagnosis. None of the 486 patients had evidence of upper tract malignancy at the time of initial diagnosis.

88% of patients received a single intravesical instillation of Mitomycin-C 40 mg following their initial TUR as per our protocol for all new non-muscle-invasive bladder tumours.

Follow-up parameters collected included recurrence rate, the time to recurrence, and rate of stage progression at recurrence. The primary endpoint of the study was to analyse the potential impact of early re-resection in detecting residual tumour and restaging disease, following initial TUR. The secondary endpoint was to assess the potential impact on tumour recurrence and stage progression. Statistical analysis was performed using SPSS version 18.0 statistical analysis software.

## Results

A total 486 consecutive patients with newly diagnosed pT1 HG-NMIBC following initial TUR of bladder tumour and a minimum follow-up period of 3 months entered the study.

In order to assess the potential impact of early re-resection, we conducted a retrospective subset analysis comparing those patients who underwent an early re-resection (Group A, *n* = 172) and those who did not (Group B, *n* = 314) (Table 1).

The mean age was 71.64 ± 8.42 years (range 44.6–91.2) and 74.72 ± 9.42 (range 39.9–100.2) in Groups A and B, respectively (*P* = 0.0087). Although there was a statistically significant difference in the age of presentation of both groups, this had no bearing on offering patients an early re-resection. The sex distribution was comparable among the two groups, with 80.2% (138/172) men and 19.8% (34/172) women in Group A and 76.1% (239/314) men and 23.9% (75/314) women in Group B (*P* = 3.089, NS). The median follow-up period for the entire cohort was 49.35 months (range 3.1–150.5). Median follow-up for Groups A and B were 50.07 months (range 3.1–141.4) and 48.98 (range 3.1–151.5) months, respectively (*P* = 0.2, NS) (Table 2).

Detrusor muscle presence in the histology specimen as a marker of TUR quality was confirmed by two cross-reporting uro-pathologists in 105/172 (61%) vs. 240/314 (76.4%) of cases in Groups A and B, respectively (*P* = 0.005).

Patients in Group A underwent an early re-resection within six weeks of initial TUR. Histological examination of the resection specimens demonstrated the presence of detrusor muscle in 77.9% (134/172) of cases.

Residual tumour was identified at re-resection in 54.6% (94/172) of patients. Among these residual tumours, 19/94 was found to be up-graded when compared to original histology, representing a rate of up-grading of 20.2%. Consequently, 12 patients in this group underwent immediate cystectomy as direct result of the histological up-staging at early re-resection.

53% (92/172) in Group A and 58.6% (184/314) in Group B received intra-vesical BCG. The recurrence rate of tumours within patients who received BCG was 50% (46/92) in Group A and 54% (99/184) in Group B (*P* = NS).

With a mean follow-up period of 53.82 ± 34.8 months, 34.8% (60/172) of patients in Group A and 42.1% (132/314) of patients in Group B developed tumour recurrence(s). The recurrence rate in those who had an early re-resection was lower than that for those who did not, although this difference was not significant (Group A 35% vs. Group B 42%, χ^2^ = 0.1561). There was no difference in recurrence-free survival between the two groups [Group A 12.21 ± 15.8 months (range 0.96–84.6) vs. Group B 13.8 ± 17.26 months (range 2.6–89.5) *P* = NS] (Table 3).

Histological assessment of those recurrences demonstrated a statistically significant difference in tumour up-staging between two groups. Of 60 cases, only two progressed in Group A compared to 19 of 132 recurrences in Group B, representing a stage progression rate at recurrence of 3.3% vs. 14.4% for Groups A and B, respectively (*P* = 0.0242) and resulting in an overall up-staging rate of 1.2% for the entire cohort in Group A (*P* = 0.0098).

## Discussion

In our study, we evaluate the potential impact of early re-resection in a cohort of 486 consecutive patients with newly diagnosed high-grade pT1 carcinoma of the urinary bladder.

The primary endpoint of this study was to assess the impact of re-resection on cancer detection rates and accurate re-staging. The rate of residual tumour after initial TUR varies widely in the published literature, ranging from 4% to 78% [2, 5, 6, 7–24], with the incidence being higher for pT1 and pTcis than in pTa tumours [13, 16, 24]. We intentionally excluded patients with pTa disease which have been evaluated by our group in a separate published study [25].

In the current study, we identified residual tumours at re-resection in 54.6% of patients in agreement with the most recent published series [24]. Twelve cases were upstaged to muscle-invasive (≥T2) disease, and these patients underwent immediate cystectomy highlighting the importance of accurate staging in the management of HG-NMIBC. Overall, 12.7% of cases were upstaged on re-resection which is similar to the rates from other published series [11, 12, 18, 19, 21] but lower that the rates reported by Mersdorf *et al* (24%) [16] and Herr (27.6%) [13]. Importantly, the study by Herr highlighted the importance of the presence of detrusor muscle in the resected specimen, with the rates of up-staging ranging from 14% to 49% in the presence or absence of muscle at first resection, respectively [13].

In our study, detrusor muscle was present in 61% (105/172 Group A, re-resection) versus 76% (240/314 Group B, no re-resection), respectively. The lower finding of muscle in Group A is not surprising as it constitutes in itself an indication for early re-resection. The main reason of the patients being offered re-resection in Group A was due to a change in the departments practice as per the EAU guidelines. There was no randomization into either arm, but the study reaffirms the role of re-resection in patients with HG-NMIBC.

At re-resection, histological examination of the specimens demonstrated the presence of detrusor muscle in 77.9% (134/172) of cases. The absence of muscle in some resections is explained by the different level of experience among surgeons performing the TUR. Zurkirchen *et al* have demonstrated the impact of the learning curve in the quality of resection, with rates of residual tumour at second resection of 37% for beginners versus 26% in the hands of experienced surgeons [23]. Similarly, Rigaud *et al* also reported a significant difference in outcomes when comparing TUR performed by senior and junior surgeons [17]. The impact of the surgeon’s skills has also been demonstrated by Brausi *et al* reviewing data from several EORTC trials [8]. In this respect, our institution is a large teaching centre, with residents at different levels of training and dexterity performing TUR. Procedures were performed by different consultants and trainees, under different levels of supervision.

The optimal interval to perform re-resection remains controversial. While Klan* et al*, reporting a rate of residual tumour of 43%, did not find any advantage in waiting more than two weeks from initial TUR [15], many studies have advocated a delay of two to six weeks, to allow post-resection inflammatory change to settle facilitating better visualization and demarcation of tissues. In our series, all patients in Group A underwent early re-resection within six weeks from initial TUR, demonstrating the presence of residual tumour in 54.6% of cases. This rate of residual disease emphasises the importance of re-resection and is similar to the 52% rate reported, also within an interval of eight weeks by Engelhardt *et al* [26]. Furthermore, it is comparable to the other published series employing shorter re-resection intervals of four to six weeks and reporting residual tumour rates ranging from 33% to 62% [7, 10, 13, 19, 20]. It therefore appears that delaying second TUR for up to six weeks does not impact negatively on the quality of the re-resection. Kohrmann *et al* [27], with an interval of up to seven weeks, found residual tumours in only 27% of cases. However, only 10% of the tumours included in that report were high grade [14]. Furthermore, in a recently published series, with a re-resection interval of up to eight weeks, Han *et al* reported the presence of residual cancer in 20/30 (66.7%) cases of pT1 tumours [12].

Interestingly, in this study, the authors reported the presence of detrusor muscle in 83.9% of re-resection specimens compared to 30.4% of initial TUR specimens. The second endpoint of this study was to evaluate the impact of early re-resection on the long-term outcome of newly diagnosed HG-NMIBCs measured as reduction in recurrence rates, increase in time to recurrence, and impact on tumour stage progression of subsequent recurrences.

We do acknowledge that our study is a retrospective analysis. However, there was a reduction in the overall recurrence rate in Group A compared to Group B (35% vs. 42% respectively), but this difference failed to reach statistical significance (*P* = 0.1561). Furthermore, there was no difference in time to the first recurrence between the two groups (Group A 12.21 ± 15.8 months vs. Group B 13.8 ± 17.26 months). These findings could possibly be explained by the fact that patients in Group B were immediately treated with a six week course of intravesical BCG prior to their first (three months) check cystoscopy.

In 2012, there is currently an acute shortage of intravesical BCG. This situation is likely to get resolved in the next few months but may arise in the future. This reinforces our recommendation for all patients with HG-NMIBC should undergo an early re-resection within six weeks of initial TURBT to prevent progression.

## Conclusion

Current European Association of Urology (EAU) guidelines recommend early re-resection in the management of HG-NMIBC. Our study confirms that early re-resection facilitates accurate staging and clearance of residual disease. Subsequent rates of tumour stage progression are significantly improved when compared to those who did not undergo early re-resection. We therefore advocate early re-resection for all patients with HG-NMIBC.

## Figures and Tables

**Table 1:** The demographic characteristics of the study groups.

	Overall cohort	Re-resection	No re-resection	*P*
Age				
Mean ± SD	73.63 ± 9.2	71.64 ± 8.41	74.72 ± 9.42	0.0087
Median	74.59	72.90	75.56	
Range	39.9–100.2	44.6 to 91.2	39.9 to 100.2	
Sex				
Male	378 (77.8%)	138 (80.2%)	239 (76.1%)	NS
Female	108 (22.2%)	34 (19.8%)	75 (23.9%)	
Follow up				
Mean ± SD	53.82 ± 34.8	56.41 ± 36.35	52.62 ± 33.74	NS
Median	49.35	50.07	48.98	
Range	3.1–150.5	3.1–141.4	3.1–150.5	
Total	486	172	314	

**Table 2:** The histological findings at initial TUR among the two study groups.

	Overall cohort	Group A	Group B
Muscle presence at first TUR			
Yes	345 (70.98%)	105 (61%)	240 (76.4%)
No	141 (29.02%)	67 (39%)	74 (23.6%)
Histology at initial TUR			
pT1 G2 high grade	148	45	103
pT1 G3	140	54	81
pTcis	35	6	29
pT1 G2 high grade + pTcis	6	0	6
pT1 G3 + pTcis	41	15	26
pT1 G2 high grade + G3	116	47	69
Total	486	172	314

**Table 3:** Histology of tumours at re-resection in Group A.

Tumour stage	Number of cases
pTx	4
pT0	74
pTcis	20
pTa	21
pT1	41
pT2	12
Total	172
